# Prevalence of Malaria and TB Coinfection at a National Tuberculosis Treatment Centre in Uganda

**DOI:** 10.1155/2019/3741294

**Published:** 2019-07-25

**Authors:** Joseph Baruch Baluku, Sylvia Nassozi, Brian Gyagenda, Margret Namanda, Irene Andia-Biraro, William Worodria, Pauline Byakika-Kibwika

**Affiliations:** ^1^Mulago National Referral Hospital, Pulmonology Division, P.O. Box 7051 Kampala, Uganda; ^2^Makerere University College of Health Sciences, Department of Internal Medicine, P.O. Box 7272 Kampala, Uganda; ^3^Makerere University Research Training Programme on Infections and Immunity (MUII-Plus), Uganda

## Abstract

The prevalence of malaria and tuberculosis (TB) coinfection is not well established in countries that are highly burdened for both diseases. Malaria could impair TB containment and increase mortality of TB patients. The objective of this study was to determine the prevalence of malaria/TB coinfection among bacteriologically confirmed adult TB patients at a national TB treatment centre in Uganda. Using a cross-sectional study design we enrolled 363 bacteriologically confirmed adult TB patients, and data on demographics and medical history was collected. Blood samples were tested for malaria blood smear, rapid malaria diagnostic test (RDT), complete blood count, haematological film analysis, HIV serology, and CD4+ and CD8+ cell counts. Malaria was defined as either a positive blood smear or RDT. The study participants were mostly male (61.4%), with a median age of 31 (interquartile range, IQR: 25-39) years, and 35.8% were HIV positive. The prevalence of malaria was 2.2% (8/363) on the overall and 5% (3/58) among participants with rifampicin resistance. A triple infection of HIV, malaria, and rifampicin resistant TB was observed in 3 participants. The prevalence of malaria among TB patients is low, and further evaluation of the epidemiological, clinical, and immunological interaction of the two diseases is warranted.

## 1. Introduction

Malaria remains a global problem with 219 million cases reported in 2017, 92% of which were in Africa [1]. Similarly, Africa has the highest incidence of tuberculosis (TB) cases at 237 per 100,000 population with 25% of the incident cases globally [2]. Uganda is among the 5 countries that contribute nearly half of the global malaria cases despite 90% of households owning insecticide treated mosquito nets [1, 3]. The geographical overlap of malaria and TB especially in areas with high incidence of HIV creates a possibility of a malaria/TB coinfection and malaria/TB/HIV triple infection [4, 5]. However, there are few epidemiological studies of malaria among TB patients. The few studies available report the prevalence of malaria among TB patients to be between 1.5 and 93.7% [6–10]. These are limited by heterogeneity considering that one of the studies used serological prevalence, two included clinically diagnosed TB patients, and another was carried out on Egyptian mummies. An attendant problem with malaria among TB patients is the overlap of symptoms, clinical signs, and complications such as anaemia, cough, and respiratory distress [11, 12]. This delays the diagnosis and initiation of treatment of either infection, yet malaria has the potential to increase mortality of TB patients [13]. The increase in mortality is plausibly due to deleterious immune interactions between TB and malaria which have not been studied widely in human subjects. Old studies among malaria/TB coinfected murine models suggested protective humoral and cellular immune responses against the complications of each disease [14, 15]. However, new evidence suggests that malaria increases mortality and morbidity of TB subjects by increasing bacillary load, hemozoin loading of macrophages, disruption of the TB granuloma, and altered T-cell responses [16–18]. TB reduces CD4 and CD8 counts and the CD4:CD8 ratio independently of malaria [19–21]. Similarly, malaria has been reported to reduce the CD4 and CD8 counts and the CD4:CD8 ratio [22, 23]. It is therefore crucial to investigate the immune effects of malaria and TB coinfection on the CD4 and CD8 cells which have a key role in TB immune responses alongside macrophages and other innate immune mechanisms [24]. This becomes imperative in the HIV/AIDS era because HIV is a major risk factor for TB reactivation by depleting CD4 cells, and malaria/TB/HIV triple infection could worsen the immune suppression [4, 25].

Understanding the prevalence, clinical factors, and immunologic interaction of malaria and TB presents an opportunity for early diagnosis, control, and eradication of two notorious diseases that present a global emergency. The primary aim of the study was to determine the prevalence of malaria/TB coinfection among bacteriologically confirmed adult TB patients at a national TB treatment centre in Uganda.

## 2. Materials and Methods

### 2.1. Study Population and Setting

We used a cross-sectional study design and consecutively enrolled 363 bacteriologically confirmed TB patients presenting to the admission facility or outpatient clinics of the Mulago Hospital tuberculosis treatment unit between August 2017 and March 2018. Mulago Hospital is the national referral hospital in Uganda located in Kampala, the capital city of Uganda. The Mulago Hospital TB unit is the national TB treatment centre for adult susceptible and drug resistant TB patients and treats 25% of the country's TB cases. Bacteriologically confirmed TB patients are referred from across the country with laboratory results for sputum microscopy, a nucleic acid amplification test (NAAT), or mycobacterial culture performed from the laboratory of the referring health facility. Bacteriologic confirmation of tuberculosis at the national TB treatment centre is performed only for patients primarily presenting for diagnostic workup at the centre unless special circumstances dictate a repeat evaluation of the sputum results from a referring health facility. We included participants aged 18 years and above, who had pulmonary bacteriologically confirmed TB by a positive sputum smear, culture, or NAAT and consented to participate in the study. Participants that had received TB treatment for 2 weeks prior to the study were excluded as TB treatment significantly reduces bacillary load and immune responses after 2 weeks [26, 27]. Study participants were identified from the TB unit and laboratory registers of the TB unit at Mulago Hospital every working day of the week and were enrolled until the desired sample size was obtained.

### 2.2. Study Measurements

A pretested structured questionnaire was administered in a face to face interview by a research assistant to obtain medical history and clinical symptoms. Seven symptoms (cough, fever, night sweats, weight loss, anorexia, headache, and chills) and their duration in days were asked of the study participants. We arbitrarily considered having 4 or more symptoms as a high symptom burden while having less than 4 symptoms was considered as a low symptom burden. The bacillary load was determined by Xpert MTB/RIF® and Ziehl-Neelsen (ZN) or Auramine sputum smear for 84% (304/363) and 14% (46/363) of study participants, respectively. This was extracted from the laboratory results from the referring health facility or the Mulago Hospital tuberculosis treatment centre laboratory. Across the country, standard techniques are employed in the laboratory diagnosis of tuberculosis according to national and World Health Organisation (WHO) recommendations [28, 29]. For 12 participants, GeneXpert was reported as “*Mycobacterium tuberculosis* (MTB) detected” while for the only participant with a culture result, it was reported as “positive”. For these 13 participants, the bacillary load grade could not be established. Bacillary load grade was standardised as shown in Table 1.

A calibrated weighing scale and stadiometer (Seca 760® and Seca 213®, respectively) were used to measure the patient's weight and height to calculate the body mass index (BMI) using the formula BMI = weight (kg)/height (meters)^2^. A BMI less than 18.5 kg/m^2^ was considered underweight, 18.5 to 24.9 kg/m^2^ was considered normal, and 25 to 29.9 kg/m^2^ was considered overweight while above 30 kg/m^2^ was considered obese. A digital thermometer (Royal Care® Model: MT 1027, SOJOY ELECTRONICS, China) was used to measure axillary temperature. A temperature of less than 35.5°C was considered as hypothermia, 35.5°C to 37.4°C as normal, above 37.4°C but less than 41.5°C as hyperthermic, and equal or above 41.5°C as hyperpyrexia.

A study nurse drew 5 milliliters of blood following standard procedures. Malaria was determined by thick and thin blood smears. A malaria antigen rapid diagnostic test (RDT) (SD BIOLINE Malaria Ag P.f/Pan®) was performed only when the smear was negative. Using RDT is justifiable as the immune effects of malaria on TB may last up to 5 months after the malaria infection has cleared [30]. One was considered to have malaria if either a thick blood smear or a rapid diagnostic test was positive. In the preparation of the thick blood smear, peripheral blood films were made from fresh EDTA blood onto clean glass slides. These were air-dried and fixed with absolute methanol for 3 minutes. They were then stained with freshly prepared Giemsa stain for 10 minutes at a pH of 6.8 before being washed off by tap water. The films were then further differentiated in buffered water (pH 6.8), washed, and air-dried. A laboratory haematologist examined the films using a binocular microscope using 100X objective. Red blood cells (RBCs) were examined to determine the type of anaemia, for hemoparasites screening and typing. White cell and red cell counts were read off the hemogram as analysed by a hemoanalyser (Sysmex® Automated haematology analyser XN series – XN 1000) at Mulago Hospital Haematology Laboratory. A hemoglobin level was read from the hemogram. It was classified according to the WHO guidelines [31] as follows: mild anaemia was 11.00 to 13 grams per decilitre (g/dl) for a male individual and 11.00 to 12 g/dl for females; moderate anaemia was 8.00 to 11 g/dl (both sexes); and severe anaemia meant hemoglobin less than 8 g/dl for both sexes. Anaemia was classified as microcytic if the mean corpuscular volume (MCV) was less than 76 femtoliters (fl) or macrocytic if MCV was above 96 fl. It was classified as hypochromic if mean cell hemoglobin (MCH) was below 24 picograms (pg). These were also read off the hemogram, and hypochromia was confirmed on peripheral film examination. CD4 and CD8 counts were measured for all study participants by flow cytometry using a flow cytometer (BD FACSCalibur™) at Makerere University joint AIDS program laboratory. The normal CD4 and CD8 counts and CD4:CD8 ranges for this study were 418-2105 cells per microliter (*μ*l), 256-1619 cells/*μ*l, and 0.52-4.1, respectively, according to the Ugandan population estimates [32]. An HIV test was performed on all participants' serum using an immunochromatographic rapid test (Alere Determine™ HIV-1/2), and a positive test was confirmed by sequential testing with another immunochromatographic test (Chembio HIV 1/2 STAT-PAK™) following the Uganda national HIV testing algorithm [33].

### 2.3. Sample Size Estimation and Statistical Analysis

The sample size estimation was informed by the prevalence of malaria among TB patients in a hospital based study in Angola using Kish Leslie formula [9, 34].

Data was entered in EpiData 3.1 and analysed using STATA 14 (StataCorp, College Station, TX, USA). The prevalence of malaria was determined as a percentage of those with malaria to the total number of study participants. Characteristics of participants with malaria/TB coinfection were summarised as frequencies and medians with corresponding interquartile ranges. The low prevalence of malaria precluded any meaningful subanalysis for significant associations of malaria/TB coinfection.

### 2.4. Ethics Approval and Consent to Participate

Study participants provided written informed consent to participate in the study. The study was approved by the Department of Internal Medicine Scientific Review Committee (SRC) and the School of Medicine Research and Ethics Committee of Makerere University College of Health Sciences (REC REF 2017-087).

## 3. Results

### 3.1. Characteristics of Study Participants

**Figure 1:**
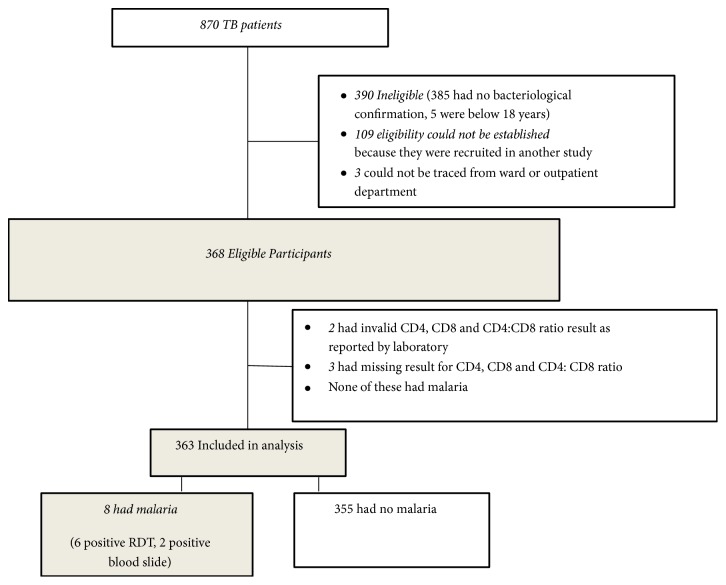
Figure showing study flow chart.

A total of 363 bacteriologically confirmed adult pulmonary TB patients were enrolled in the study. The study flow diagram is shown in Figure 1.

Majority of the participants, 61.4% (223/363), were male; were from urban areas, 67.5% (245/363); and had at least primary level of education, 93.1% (338/363). The median age was 31 (IQR: 25-39) years and 35.8% (130/363) of the participants were HIV positive. Of the HIV positive participants, 77% (97/130) were taking cotrimoxazole prophylaxis while 49.2% (64/130) were taking antiretroviral therapy (ART). History of TB treatment was found in 15.2% (55/363), of whom 60.1% (38/55) reported having received treatment less than 3 years prior to enrolment in the study. The majority (67.5% (245/363)) of participants reported sleeping under a mosquito net the previous night. History of indoor residual spraying of accommodation units or houses in the last six months was reported by 9.4% (34/363) of the participants. History of malaria treatment in the last 1 month prior to the study was reported by 20.7% (75/363) of study participants. Anaemia was found in 58.7% (210/358) of participants. Hypochromia was found in 70.4% (252/358) of participants, microcytosis in 40.5% (145/358), and macrocytosis in 8.4% (30/358). The proportions of participants reporting cough, weight loss, night sweats, fever, chills, headache, and anorexia were (n=363) 98.1%, 84.0%, 69.1%, 60.1%, 36.1%, 32.2%, and 26.2%, respectively. Other characteristics of study participants are summarised in Table 2.

### 3.2. Prevalence of Malaria among TB Patients

The study found a prevalence of malaria/TB coinfection of 2.2% (8/363) among study participants. Six (75%) of these had a positive malaria antigen test (RDT) while 2 (25%) had a positive blood smear. All participants with malaria had* Plasmodium falciparum*. Alongside one with a positive RDT, the 2 participants with a positive malaria blood smear were HIV positive and had rifampicin resistance. Thus, we found a prevalence of malaria/TB/HIV triple infection of 0.83% (3/363) among the study participants.  Table 3 shows the prevalence of malaria in the study.

### 3.3. Characteristics of Participants with Malaria and TB Coinfection

We observed that participants with malaria/TB coinfection were mostly male (62.5%) and less than 35 years of age (87.5%) with no history of TB treatment (87.5%). The proportions of malaria/TB coinfected participants with cough, night sweats, fever, weight loss, chills, anorexia, and headache was 100%, 87.5%, 75%, 75%, 62.5%, 50%, and 37.5%, respectively, with a respective median duration of 48.5 (IQR: 18.5-60), 7 (IQR: 2-30), 22 (IQR: 8-30), 25.5 (IQR: 15-60), 7 (IQR: 7-14), 5.5 (IQR: 2-19.5), and 14 (IQR: 7-30) days. The median CD4 and CD8 cell counts and CD4:CD8 were 864 (IQR: 257-1281) cells/*μ*l, 527 (IQR: 443-740) cells/*μ*l, and 1.82 (IQR: 1.51-1.99), respectively. The current use of a mosquito net and history of indoor residual spraying, smoking, alcohol use, and malaria treatment in preceding month were 87.5%, 12.5%, 25.0%, 62.5%, and 25%, respectively.  Table 4 shows other characteristics of participants with malaria/TB coinfection.

## 4. Discussion

This study aimed at determining the prevalence of malaria and TB coinfection among bacteriologically confirmed adult TB patients. The study found a low prevalence of malaria among adults with bacteriologically confirmed TB patients. This could be explained by increased production of gamma interferon (IFN-*γ*), tumor necrosis factor alpha (TNF-*α*), and humoral factors induced by tuberculosis infection that are protective against malaria infection [14, 15, 35]. The observed low prevalence could also be due to the demographic characteristics of our study population. Our study population was an adult population from predominantly urban settings which inherently have a low prevalence of malaria [3, 36]. Further, 20% of the study participants reported a history of malaria treatment in the previous month. This too could be contributory to the low prevalence. All participants with malaria in our study had* Plasmodium falciparum*. This is expected as* P. falciparum* is the predominant* Plasmodium* species found in Uganda accounting for 97% of malaria species [3]. Other hospital based studies also found a low prevalence of malaria/TB coinfection as well. Anyangwe et al. (2016) found a prevalence of 1.5% in Cameron and Range et al. (2007) found it at 4.3% in Tanzania. An exception is Valadas et al. (2007) that found a prevalence of 37.5% in Angola. However, their study was retrospective and included children and clinically diagnosed TB patients, and this could have overestimated the prevalence. To the best of our knowledge, our study findings are the first to report malaria among drug resistant TB (DR-TB) patients. The relationship between DR-TB and malaria is yet to be defined and thus an interesting area for future research.

A triple infection of HIV, malaria, and DR-TB was observed in our study and we have not found other reports on the same. However, almost 20% of our study population with a GeneXpert had rifampicin resistance. Therefore, the observation of DR-TB and malaria coinfection as well as malaria/HIV/DR-TB triple infection may be coincidental. Nevertheless, DR-TB patients have high CD4 cell counts that are predominantly regulatory T-lymphocytes which reduce the expression of IFN-*γ* in these patients [37–39]. There is a need for further evaluation of the effect of these immune responses on the susceptibility of DR-TB patients to other infections such as malaria. Moreover, the qualitative and quantitative effects of a malaria/HIV/DR-TB triple infection on the CD4 cells are unknown. Although they did not evaluate for drug resistance, other studies in African settings report a higher prevalence of malaria/TB/HIV triple infection than ours (0.83%): 2.5% in Cameroon [6], 3.9% in Nigeria [4], and 6.0% in Tanzania [7]. The most recent of these studies by Anyangwe et al. (2016) found a lower prevalence (2.5%) than the older studies and Range et al. studied an HIV positive population. Not only has the HIV burden declined across sub-Saharan Africa over the years, but also West African countries like Nigeria have a higher prevalence of HIV than Uganda [40]. However, 77% of the HIV positive participants in our study were taking cotrimoxazole prophylaxis which confers protection against malaria [41]. This may explain why our prevalence of malaria/TB/HIV is lower than previous studies.

Our study had some limitations. The study was carried out at a national TB treatment referral centre and this limits generalisability of results due to referral bias. However, we found a prevalence of malaria that is comparable to other settings, and it seems that the choice of the study site have not affected the findings. The use of different laboratory results for bacillary load from different laboratories could have posed a limitation of standardisation of reporting smear results by different microscopists. However, 84% of our study participants had TB diagnosed by GeneXpert® which has standard reporting of bacillary load grade. We believe that standardisation of bacillary load did not affect our results significantly. Lastly, our study was not powered to explore associated factors. The small number of participants with malaria/TB coinfection limited any meaningful subgroup analyses.

## 5. Conclusion

The prevalence of malaria among patients with bacteriologically confirmed TB is low. A further evaluation of the associated factors is warranted.

## Figures and Tables

**Table 1:** Grading of bacillary load by laboratory result.

Bacillary Load Grade	Smear	Xpert MTB/RIF®
	(ZN^#^ or Auramine stain)	(GeneXpert)^*β*^
Very Low	Scanty (1-9AFBs^¥^/100 fields)	Very Low
Low	1+ (1-9AFBs/10 fields)	Low
Medium	2+ (1-10/field)	Medium
Very High	3+ (>10/field)	High

#: ZN: Ziehl-Neelsen.

*β*: Bacillary load by Xpert MTB/RIF® is determined by cycle threshold values.

¥: AFBs: acid fast bacilli.

**Table 2:** Characteristics of study participants.

Participant Characteristics	Frequency (n=363)	Percentage (%)
*History of smoking*		
Never	258	71.1
Past smoker (≥6 months ago)	50	13.8
Current smoker (<6 months ago)	55	15.2
*Alcohol usage*		
Never	176	48.5
Past alcohol use (≥6 months ago)	58	16.0
Current alcohol use (<6 months ago)	129	35.5
*Bacillary Load Grade (n = 351)*		
Very Low	47	13.4
Low	81	23.1
Medium	121	34.5
Very High	102	29.1
*Rifampicin Resistance (n=304)*		
Detected	58	19.1
Indeterminate	1	0.3
*Symptom Burden*		
<4 symptoms (Low)	134	36.9
4-7 Symptoms (High)	229	63.1
*Body Mass Index (BMI)*		
Underweight	188	51.8
Normal	158	43.5
Overweight	14	3.9
Obese	3	0.8
*Temperature (n=361)*		
Hypothermic	120	33.2
Normal	193	53.5
Hyperthermic	48	13.3
*Red Blood Cell Count* ^*1*^		
<Lower Limit of Normal (LLN)	80	22.0
Normal	257	70.8
>Upper Limit of Normal (ULN)	26	7.2
*White Blood Cell Count* ^*1*^ * (n=357)*		
<LLN	11	3.1
Normal	237	66.4
>ULN	109	30.5
*Anemia (n=358)*		
Severe Anemia	24	6.7
Moderate Anemia	101	28.2
Mild Anemia	85	23.7
Normal Hemoglobin	148	41.3

^1^Ranges for cell counts are for adult Ugandans [42].

**Table 3:** Prevalence of malaria among patients with TB.

	Frequency of malaria	Prevalence (%)	95% confidence interval
Total population (n=363)	8	2.20	(1.10-4.35)
Rifampicin resistance (n=58)	3	5.17	(1.62-15.30)
TB/HIV co-infection (n=130)	3	2.31	(0.74-7.00)
TB without HIV( n=233)	5	2.15	(0.89-5.08)

**Table 4:** Characteristics of participants with malaria/TB co-infection.

Characteristics of participants with malaria/TB co-infection	Frequency (n = 8)	Percentage (%)
*Symptom burden *		
Low	2	25.0
High	6	75.0
*Body mass Index*		
low	3	37.5
Normal	5	62.5
*Temperature*		
Hypothermia	1	12.5
Normal	5	62.5
Hyper thermic	2	25.0
*Bacillary Load Grade*		
Very Low	2	25.0
Low	1	12.5
Medium	3	37.5
Very High	2	25.0
*Rifampicin resistance (n = 7)*	3	42.9
*Anemia*		
Moderate	1	12.5
Mild	4	50.0
Normal hemoglobin	3	37.5
*MCH*		
Normal	7	87.5
Hypochromic	1	12.5
*MCV*		
Microcytic	6	75.0
Normal	2	25.0

## Data Availability

Datasets generated and/or analysed during this study are available from the corresponding author upon reasonable request.

## References

[1] World Health Organization (2018). *World Malaria Report*.

[2] World Health Organization (2018). *Global Tuberculosis Report*.

[3] Ministry of Health (2015). *Uganda Malaria Indicator Survey 2014-2015*.

[4] Audu R. A., Onwujekwe D. I., Onubogu C. C. (2005). Possible impact of co-infections of tuberculosis and malaria on the CD4+ cell counts of HIV patients in Nigeria. *Annals of African Medicine*.

[5] Bhutta Z. A., Sommerfeld J., Lassi Z. S., Salam R. A., Das J. K. (2014). Global burden, distribution, and interventions for infectious diseases of poverty. *Infectious Diseases of Poverty*.

[6] Anyangwe I. (2016). The effect of Malaria/HIV/TB triple infection on malaria parasitaemia, haemoglobin levels, CD4+ cell and acid fast bacilli counts in the south west region of Cameroon. *Journal of Infectious Pulmonary Diseases ( ISSN 2470-3176 )*.

[7] Range N., Magnussen P., Mugomela A. (2007). HIV and parasitic co-infections in tuberculosis patients: A cross-sectional study in Mwanza, Tanzania. *Annals of Tropical Medicine and Parasitology*.

[8] Lalremruata A., Ball M., Bianucci R. (2013). Molecular identification of *Falciparum malaria* and human tuberculosis co-infections in mummies from the Fayum Depression (Lower Egypt). *PLoS ONE*.

[9] Valadas E., Gomes A., Sutre A. (2013). Tuberculosis with malaria or HIV co-infection in a large hospital in Luanda, Angola. *The Journal of Infection in Developing Countries*.

[10] Adebajo A. O., Smith D. J., Hazleman B. L., Wreghitt T. G. (1994). Seroepidemiological associations between tuberculosis, malaria, hepatitis B, and AIDS in West Africa. *Journal of Medical Virology*.

[11] Bahbahani H., Al-Rashed M., Almahmeed M. (2014). Tuberculosis and autoimmune hemolytic anemia: Case report and literature review. *Journal of Applied Hematology*.

[12] Anstey N., Jacups S., Cain T. (2002). Pulmonary Manifestations of Uncomplicated Falciparum and Vivax Malaria: Cough, Small Airways Obstruction, Impaired Gas Transfer, and Increased Pulmonary Phagocytic Activity. *The Journal of Infectious Diseases*.

[13] Colombatti R., Penazzato M., Bassani F. (2011). Malaria prevention reduces in-hospital mortality among severely ill tuberculosis patients: a three-step intervention in Bissau, Guinea-Bissau. *BMC Infectious Diseases*.

[14] Page K. R., Jedlicka A. E., Fakheri B. (2005). Mycobacterium-Induced Potentiation of Type 1 Immune Responses and Protection against Malaria Are Host Specific. *Infection and Immunity*.

[15] Murphy J. R. (1980). Host defenses in murine malaria: Immunological characteristics of a protracted state of immunity to Plasmodium yoelii. *Infection and Immunity*.

[16] Li X., Zhou X. (2013). Co-infection of tuberculosis and parasitic diseases in humans: a systematic review. *Parasites & Vectors*.

[17] Mueller A., Behrends J., Hagens K. (2012). Natural transmission of plasmodium berghei exacerbates chronic tuberculosis in an experimental co-infection model. *PLoS ONE*.

[18] Hawkes M., Li X., Crockett M. (2010). Malaria exacerbates experimental mycobacterial infection in vitro and in vivo. *Microbes and Infection*.

[19] Hussain T., Kulshreshtha K. K., Yadav V. S., Katoch K. (2015). Cd4+, Cd8+, Cd3+ Cell Counts And Cd4+/cd8+ ratio among patients with mycobacterial diseases (leprosy, Tuberculosis), Hiv infections, and normal healthy adults: a comparative analysis of studies in different regions Of India. *Journal of Immunoassay and Immunochemistry*.

[20] Davoudi S., Rasoolinegad M., Younesian M. (2008). CD4+ cell counts in patients with different clinical manifestations of tuberculosis. *The Brazilian Journal of Infectious Diseases*.

[21] Yin Y., Qin J., Dai Y. (2015). The CD4+/CD8+ ratio in pulmonary tuberculosis: systematic and meta-analysis article. *Iranian Journal of Public Health*.

[22] Lisse I. M., Aaby P., Whittle H., Knudsen K. (1994). A community study of T lymphocyte subsets and malaria parasitaemia. *Transactions of the Royal Society of Tropical Medicine and Hygiene*.

[23] Kassa D., Petros B., Mesele T., Hailu E., Wolday D. (2006). Characterization of peripheral blood lymphocyte subsets in patients with acute plasmodium falciparum and p. vivax malaria infections at wonji sugar estate, Ethiopia. *Clinical and Vaccine Immunology*.

[24] Prezzemolo T., Guggino G., la Manna M. P., di Liberto D. D., Dieli F., Caccamo N. (2014). Functional signatures of human CD4 and CD8 T cell responses to Mycobacterium tuberculosis. *Frontiers in Immunology*.

[25] Ellis P. K., Martin W. J., Dodd P. J. (2017). CD4 count and tuberculosis risk in HIV-positive adults not on ART: a systematic review and meta-analysis. *PeerJ*.

[26] Bouti K., Aharmim M., Marc K. (2013). Factors influencing sputum conversion among smear-positive pulmonary tuberculosis patients in Morocco. *ISRN Pulmonology*.

[27] Su W., Perng W., Huang C. (2010). Association of reduced tumor necrosis factor alpha, gamma interferon, and Interleukin-1 *β* (IL-1*β*) but increased IL-10 expression with improved chest radiography in patients with pulmonary Tuberculosis. *Clinical and Vaccine Immunology*.

[28] Lumb R., Van Deun A., Bastian I., Fitz-Gerald M., Pathology S. A. (2013). *Diagnosis of Tuberculosis by Sputum Microscopy: The Handbook*.

[29] Ministry of Health (2018). *TB Laboratory Network Manual*.

[30] Blank J., Eggers L., Behrends J., Jacobs T., Schneider B. E. (2016). One episode of self-resolving Plasmodium yoelii infection transiently exacerbates chronic mycobacterium tuberculosis infection. *Frontiers in Microbiology*.

[31] World Health Organisation (2011). *Haemoglobin concentrations for the diagnosis of anaemia and assessment of severity. Concentrations en hémoglobine permettant de diagnostiquer l’anémie et d’en évaluer la sévérité*.

[32] Nanzigu S., Waako P., Petzold M. (2011). CD4-T-lymphocyte reference ranges in Uganda and its influencing factors. *LabMedicine*.

[33] Ministry of Health (2016). *National HIV Testing Services Policy and Implementation Guidelines*.

[34] Kish L. (1995). *Survey Sampling. Revised edition*.

[35] Perez-Mazliah D., Langhorne J. (2014). CD4 T-cell subsets in malaria: Th1/Th2 revisited. *Frontiers in Immunology*.

[36] Yegorov S., Galiwango R. M., Ssemaganda A. (2016). Low prevalence of laboratory-confirmed malaria in clinically diagnosed adult women from the Wakiso district of Uganda. *Malaria Journal*.

[37] Geffner L., Yokobori N., Basile J. (2009). Patients with multidrug-resistant tuberculosis display impaired Th1 responses and enhanced regulatory T-cell levels in response to an outbreak of multidrug-resistant Mycobacterium tuberculosis M and Ra strains. *Infection and Immunity*.

[38] Sun E., Xia D., Li B. (2017). Association of immune factors with drug-resistant tuberculosis: a case-control study. *Medical Science Monitor*.

[39] Pinheiro R. O., de Oliveira E. B., dos Santos G. (2013). Different immunosuppressive mechanisms in multi-drug-resistant tuberculosis and non-tuberculous mycobacteria patients. *Clinical & Experimental Immunology*.

[40] Kharsany A. B. M., Karim Q. A. (2016). HIV infection and AIDS in sub-Saharan Africa: current status, challenges and opportunities. *The Open AIDS Journal*.

[41] Kasirye R. P., Baisley K., Munderi P. (2016). Incidence of malaria by cotrimoxazole use in HIV-infected Ugandan adults on antiretroviral therapy. *AIDS*.

[42] Eller L. A., Eller M. A., Ouma B. (2008). Reference intervals in healthy adult ugandan blood donors and their impact on conducting international vaccine trials. *PLoS ONE*.

